# Impact of a binary triage system and structural reorganization of emergency department on health care workers exposed to suspected COVID-19 patients—a single-centre analysis

**DOI:** 10.1186/s12245-021-00384-3

**Published:** 2021-09-23

**Authors:** Mahathar Abd. Wahab, Sufian Safaai, Ismail Mohd Saiboon

**Affiliations:** 1 Emergency and Trauma Department, Kuala Lumpur Hospital, Jalan Pahang, 50586 Kuala Lumpur, Malaysia; 2 Department of Emergency Medicine, Faculty of Medicine, Universiti Kebangsaan Malaysia (UKM), Jalan Yaacob Latif Bandar Tun Razak, Cheras, 56000 Kuala Lumpur, Malaysia

**Keywords:** COVID-19, Health care worker, Triage system, Emergency department structure

## Abstract

**Background:**

A binary triage system based on infectivity and facilitated by departmental restructuring was developed to manage suspected COVID-19 patients with an aim to provide effective prevention and control of infection among health care workers (HCWs) in the emergency department. This study analyses the effectiveness of the new triage system and structural reorganization in response to the COVID-19 pandemic.

**Methods:**

A cross-sectional observational study was conducted in the Emergency and Trauma Department, Hospital Kuala Lumpur (ETDHKL). The implementation of a binary triage system separates patients with risk of COVID-19 who present with fever and respiratory symptoms from other patients. Data on exposed HCWs to COVID-19 patients were captured pre-restructuring and post-restructuring of the emergency department and analysed using descriptive statistics.

**Results:**

A total of 846 HCWs were involved in this study. Pre-restructuring reported 542 HCWs exposed to COVID-19 patients while post-restructuring reported 122. Using the four categorical exposure risks for HCWs which are no identifiable risk, low risk, medium risk, and high risk, the number of HCWs exposed during pre-restructuring were 15(1.8%), 504 (59.6%), 15 (1.8%), and 8 (0.9%), respectively, while post-restructuring the numbers were 122 (14.4%), 8 (0.9%), 109 (12.9%), and 5 (0.1%), respectively. There was a 77.5% reduction in the number of exposed HCWs after our implementation of the new system (542 vs 122).

**Conclusion:**

A binary triage system based on severity and infectivity and supported with structural reorganization can be effective in reducing HCWs COVID-19 exposure.

## Introduction

The world was on high alert when Wuhan city in China was hit hard by a novel coronavirus infection, severe acute respiratory syndrome coronavirus 2 (SARS-CoV-2), in late December 2019. During the time of the initial outbreak in Wuhan, the Emergency and Trauma Department, Hospital Kuala Lumpur (ETDHKL) started a contingency plan to face this threat of a pandemic. Malaysia is a country strategically located in the heart of Southeast Asia, and the centre of attraction among Chinese tourists. Malaysia was struck by the first wave of novel coronavirus infections starting on the 24th of January 2020. The second wave of infections ensued following contributions by local clusters in late February 2020 [1, 2].

Reports of COVID-19 infections among health care workers (HCWs) started to emerge in Wuhan, China in January 2020 [3]. A single-centre study by Xiaoqian Lai et al. [4] reported 1.1% of infections among HCWs of Tongji Hospital, Wuhan, China, whereby 0.9% of the HCWs with COVID-19 infections were asymptomatic. The most significant infective source was from COVID-19 patients (59.1%). A similar study conducted by Kluytmans-van den Bergh MFQ et al. [5] in Dutch hospitals reported an infection rate of 1% among HCWs. In our local settings, a study by University Malaya Medical Centre reported a prevalence of 0.3% COVID-19 infection among HCWs [6]. On March 20th, 2020, the Ministry of Health (MOH) Malaysia reported the first case of a HCW in Malaysia who was a trainee nurse at a private hospital becoming infected with COVID-19 [7].

World Health Organization (WHO) published an investigation protocol to help health institutions gather crucial data on COVID-19 exposure among HCWs [8]. Standardized data collection among health institutions aids researchers in understanding the spread, severity, spectrum of disease, and impact of COVID-19 on healthcare systems and the community. Guidelines for the management of exposed and infected HCWs were published to help hospitals to contain the spread of COVID-19 infections among HCWs while maintaining an adequate workforce to run the hospital’s departments. According to WHO, exposure to COVID-19 patients is defined as close contact (within 1 m distance and for more than 15 min) with a suspected/probable/confirmed COVID-19 patient(s) or indirect contact with fomites (for example, clothes, linen, utensils, furniture and so on) or with materials, devices or equipment linked to a suspected/probable/confirmed COVID-19 patient(s) [8].

Malaysia established a guideline called the Management of Healthcare Worker During COVID-19 Pandemic Outbreak [9]. This guideline thoroughly details risk assessment, potential exposure criteria, management of HCWs exposed to COVID-19, and crisis strategies. The guideline divides the risk exposure into four categories according to the circumstances and the practices of HCWs during the handling of COVID-19 patients as shown in Table 1.

**Table 1:** Ministry of Health Malaysia category of risk exposure of COVID-19 for HCW

Category of risk exposure	Circumstances
High-risk exposures	• HCW who performed or were present in the room for procedures that generate aerosols or during which respiratory secretions are likely to be poorly controlled* on patients with COVID-19 AND • When the healthcare providers’ eyes, nose, or mouth were not protected.
Medium-risk exposures	• HCW who had prolonged close contact with a confirmed COVID-19 case, AND • where HCW mucous membranes or hands were exposed to potentially infectious materials for COVID-19
Low-risk exposures	• Any inconsistencies in adherence to PPE while in close contact with a confirmed COVID-19 case
No identifiable risk	• HCW without direct close contact with a confirmed COVID-19 case • No entry into active patient’s area • HCW who adhere to recommended PPE

Source: Ministry of Health, Malaysia. 2020

All patients with respiratory symptoms who present to the emergency department (ED) initially go through a triaging process before being treated. The conventional ED triage system is traditionally based on the severity of the patients. With the evidence that COVID-19 has high infectivity rates, and most patients present with only mild respiratory symptoms [10–12], HCWs at the ED were unknowingly at risk as they were at the frontlines. Ministry of Health (MOH) Malaysia reported on the 23rd April 2020 that a total of 325 HCWs were infected with COVID-19 which was 5.8% of the total of 5603 positive cases [13]. However, the majority of these cases contracted the disease from the community via local transmissions.

Suspected COVID-19 patients who presented to ED were triaged via two different pathways (binary triage system). The first pathway was for ambulatory patients that have the epidemiological link and risk of COVID-19. Patients that were triaged through this pathway were managed in a dedicated COVID-19 screening centre. The second pathway was for the unstable patients that presented with respiratory symptoms, but had no or uncertain epidemiological links, and these undifferentiated patients were triaged and managed in the resuscitation zone. For protection, HCWs working in both of these zones wore full personal protective equipment (PPE). However, Nguyen LH et al. [14] reported that having adequate PPE is not enough to prevent the infection transmission from the patients to HCWs. Moreover, widespread wearing of PPEs by all the emergency personnel was uneconomical in times where PPEs are limited and costly. Therefore, changes of the triage system, structure, and design were also needed to be implemented to manage risks of COVID-19 exposure to HCWs.

We conducted an observational study on the impact of the ED's binary triage system together with departmental structural reorganization by comparing available data before and after the changes were carried out. We hypothesized that the introduction of a binary triage system and structural reorganization which were more relevant to the current pandemic situation will reduce the risk of HCWs getting infected by COVID-19-positive patients.

## Methods

This is a cross-sectional observational study conducted in the ETDHKL, Kuala Lumpur Federal Territory, Malaysia. The study was carried out from the 5th of May to the 31st of July 2020. The data was collected from 27th of February to the 26th of April 2020 for 60 days and the 28th March to the 26th April was set as the comparative period. Since this is an observational study, universal sampling was performed. The number of ED staff during the study period was taken as the population for the study. This study was approved by the research and ethics committee of the National Medical Research Register and the research number is NMRR-20-2636-57197.

### The restructuring

**Fig. 1:**
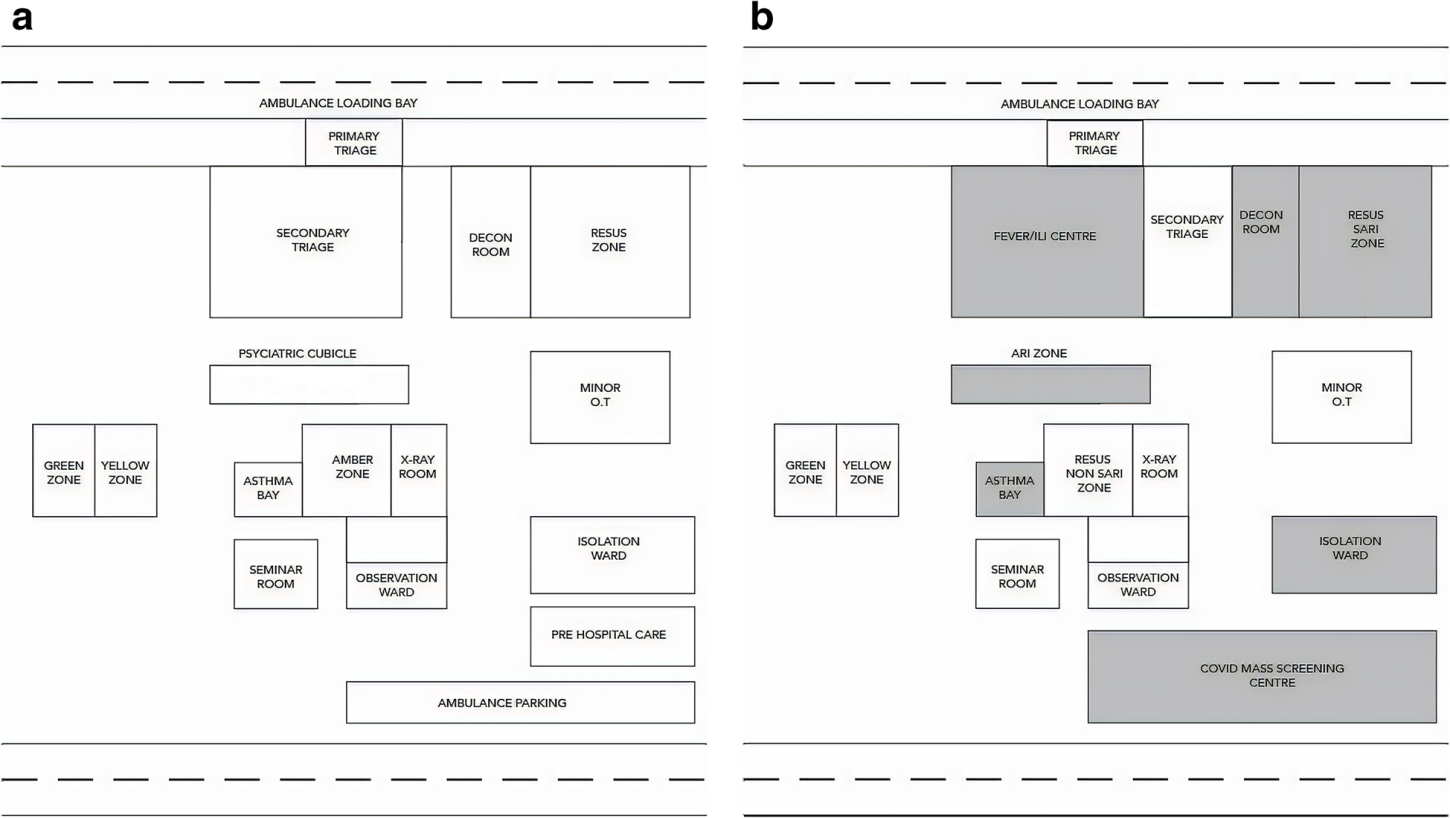
**A** Conventional Triage Zones prior to COVID-19 outbreak. **B** New triage zones during COVID-19 pandemic

The traditional structure of the ED based on the Malaysian Triage Category [15] includes the critical zones (zone I and II), semi-critical zones, and non-critical zones supported by asthma bay, intermediate care ward, psychiatric assessment bay, decontamination room and isolation ward (Fig. 1A). The pre-restructuring triage criteria for the respective zones are shown in Table 2. The structural reorganization of the ED divides the department into two distinct areas which are the ‘clean areas’ and ‘dirty areas.’ Dirty areas consists of areas for severe acute respiratory illness (SARI), influenza-like illness (ILI), a decontamination room, an isolation ward, and a COVID mass screening area (CMSA) (Fig. 1B). The Critical zone I was reorganized as SARI for managing unstable SARI patients. The new ILI area was created to manage ambulating and stable patients presenting with symptoms of upper respiratory tract infection. The decontamination room was dedicated to managing the unstable patients who were confirmed to have the COVID-19 infection or those patients under investigation (PUI). The negative pressure isolation ward equipped with 12 beds acts as a short stay ward for SARI patients awaiting admission to the allocated SARI wards. CMSA is a dedicated mass screening area separated from the main ED, it operates 24 h/day, and serves as an assessment and treatment area for the ambulatory and stable patients who fulfil the PUI criteria for COVID-19 infections. The remainder areas are the clean areas (Fig. 1B).

**Table 2:** ETDHKL pre-restructuring triage criteria

Treatment zones	Triage criteria
Red zone (Critical zone I )	• Cardiac arrest • Respiratory arrest • Compromised Airway (A), Breathing (B) or Circulation (C)—need immediate lifesaving procedures. • Apply to all cases a. Any cases involving airway compromise, triage to red zone b. Any cases involving two or more systems with or without airway compromise, triage to red zone
Amber zone (Critical zone II )	• Stable airway • Patients requiring oxygen support including non-invasive ventilation (NIV) • Patients that require fluid resuscitation including patients in compensated shock with or without inotropic support
Yellow zone (Semi-critical zone)	Patients with stable airway, breathing and circulation but in moderate pain and unable to ambulate.
Green zone (Non-critical zone)	• Patients with stable airway, breathing, circulation and no alteration of mental status. • Ambulating unaided or with a wheelchair. • Green zone patients are further divided into sub-categories: a. Green category 1 (Fast lane) – seen less than 10 min b. Green category 2 (require initial management /first aid treatment before seen by a doctor) c. Green category 3 (other than the above)
Decontamination room	• Can accommodate any patient with haemodynamic compromise including those requiring intubation and resuscitation. • All highly infectious diseases a. Meningococcaemia b. Middle East respiratory syndrome coronavirus c. Ebola d. Diphtheria e. Measles • All high lethality environmental toxin exposure a. Paraquat b. Organophosphate • All HAZMAT cases - Hazardous materials including exposure to chemicals, nuclear waste products, biological and radiological agent

Source: ETDHKL Triage Manual

**Fig. 2:**
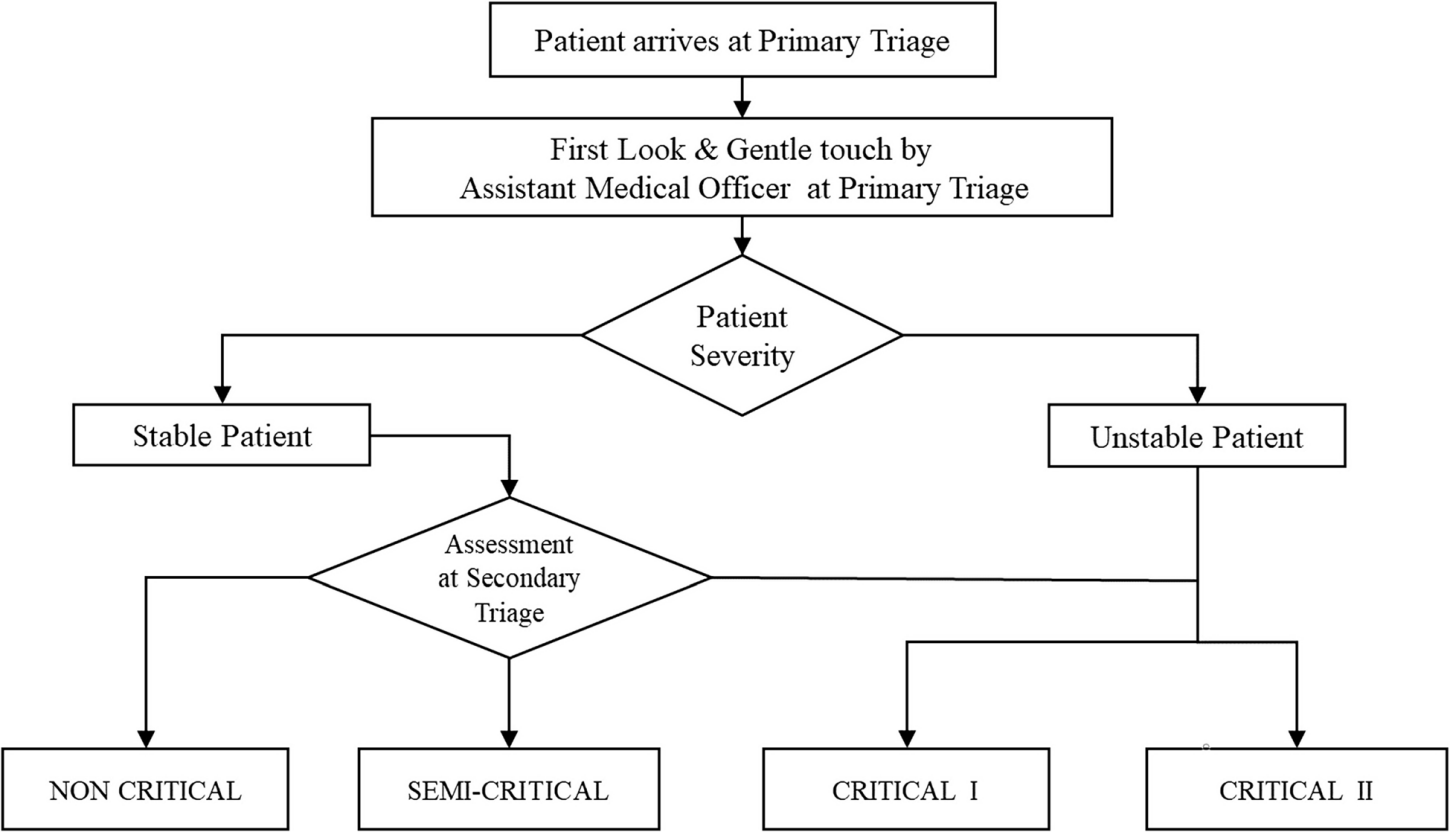
Conventional triage workflow

**Fig. 3:**
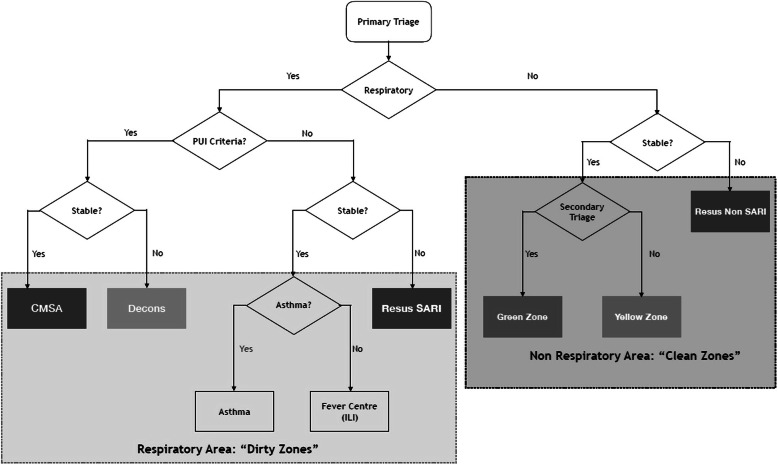
New triage algorithm with dirty zones and clean zones

**Fig. 4:**
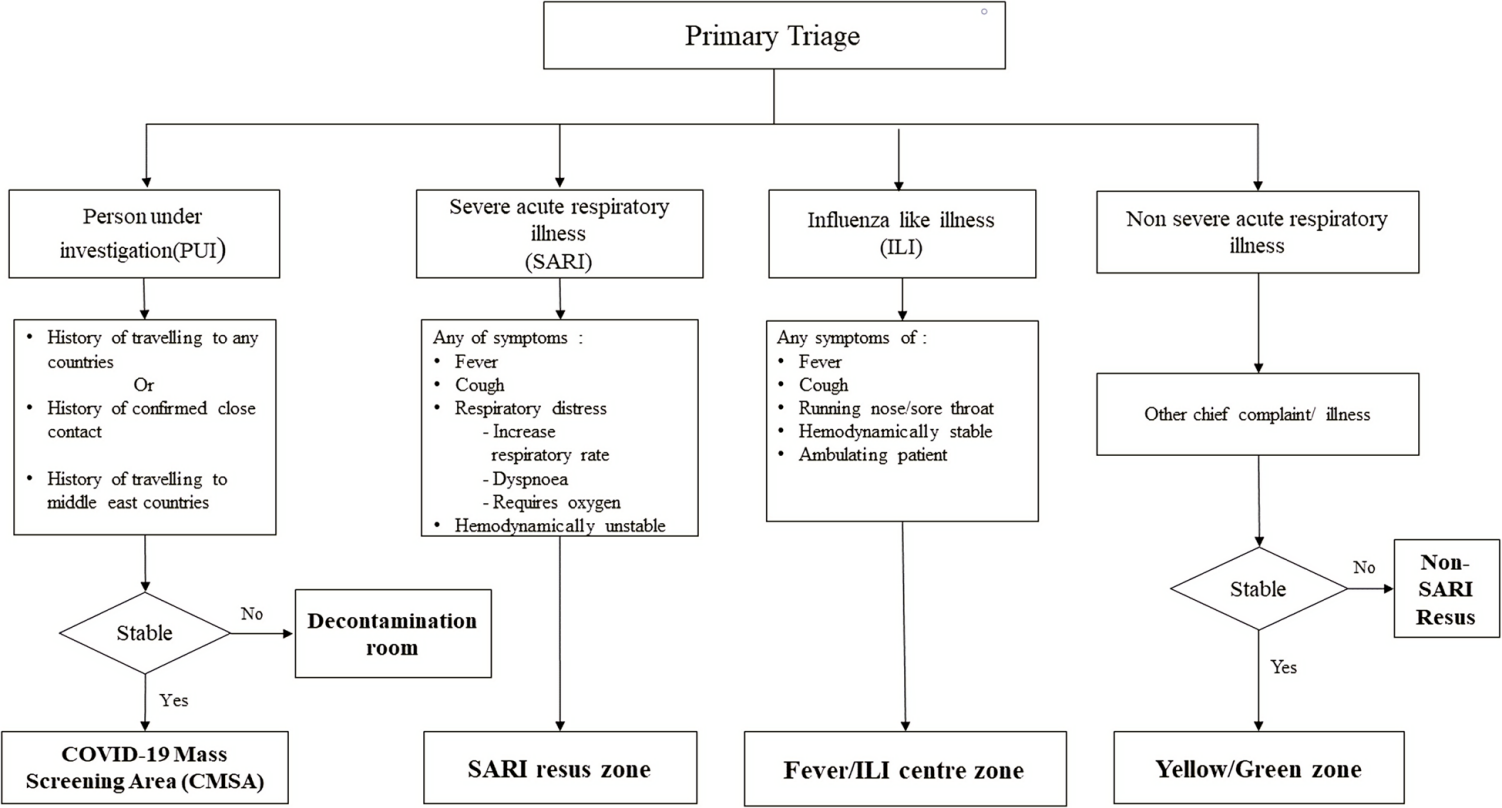
New triage criteria during COVID-19 pandemic

The purpose for introducing the new triage system was due to the fact of the undifferentiated presentations of infected COVID-19 patients from those with other diseases. The conventional way of the triage workflow (Fig. 2) was changed to the binary triage system workflow (Fig. 3). The triage system was targeted to separate the patient’s flow into dirty or clean areas. The new triage criteria divide the patients into PUI, SARI, ILI, or non-respiratory illness at the primary triage level (Fig. 4).

A comprehensive policy on the use of PPE was implemented in both dirty and clean areas. For dirty areas, clinical staff are required to wear hospital scrubs with full PPE which consists of disposable cap, disposable face shield, surgical mask, disposable sterile gown, disposable plastic apron, and disposable gloves when managing patients. When treating confirmed COVID-19-positive patients or performing aerosol-generating procedures such as intubation, cardiopulmonary resuscitation (CPR), and Ryle’s tube insertion, HCWs are obligated to wear a N95 mask. In the clean areas, HCWs must wear a surgical mask with a face shield at all times and a disposable plastic apron for additional protection during procedures. By enforcing these measures, the ED was able to maintain a steady supply of PPE in the department. The lower level of PPE at the clean areas was relatively safe as asymptomatic COVID-19 patients have a lower risk of transmission of the infection to HCWs [16].

To address the asymptomatic patients with COVID-19 infections or atypical presentations apart from respiratory symptoms, risk stratifications were done during the first contact at the triage counter. At the triage counter, a detailed history regarding the epidemiological link was obtained, and the patients were required to sign a declaration form. This is to identify the patients from the high-risk group which come from an active COVID-19 cluster, or red zone area with high prevalence of active cases. The patients that exhibit those risks will be managed as ‘infective’ patients.

The restructuring of ED also involves the allocation of a dedicated area for the donning and doffing of PPE next to the dirty areas and a restroom equipped with a shower for clinical staff. A special task force was created for regular training of the proper techniques of donning and doffing of PPE. This task force was also in charge of supervising the strict adherence of the HCWs to COVID-19 Standard Operating Procedures at the workplace. New policies were enforced such as the requirement for all the patients to wear a face mask at all times and aseptic hand washing before and after contact with any patients in all zones. Clear demarcation lines on the floor of the ‘dirty’ treatment zones were created between the patient’s area and the HCW workstations to prevent contamination of equipment, patient’s case files and the HCW workstation itself. The demarcation lines also functioned as areas for staff to wear or remove their gloves and plastic aprons before and after seeing the patients. Social distancing between staff was advocated at all times and continuous medical education lectures were switched to online platforms.

### Study protocol

In our study, HCWs were defined as any staff in the health care facility involved in the provision of care for a COVID-19 patient, including those who have been present in the same area as the patient as well as those who may not have provided direct care to the patient but who have had contact with the patient’s body fluids, potentially contaminated items, or environmental surfaces. This is in accordance to the definition set out by the WHO. The inclusion criteria are all HCWs who are working in the ETDHKL who have direct contact with the patients. The exclusion criteria for this study are HCWs exposed to or had contracted the COVID-19 infection from close contacts other than the patients that presented to the ETDHKL within the last 14 days, and HCWs who attended to patients at COVID-19 Mass Screening Area (CMSA) with full PPE and practised maximum precautions.

All the reported cases of COVID-19-positive patients that presented to the ETDHKL were traced over 30 days before and after the implementation of a new triage system. HCWs on duty during handling of the respective COVID-19 patients were traced and interviewed by the Emergency Department Infection Control committee to determine the categories of risk exposure based on MOH protocol. Health care assistants, staff nurses, assistant medical officers (AMO), and doctors including the emergency physician, medical officer, and house officer who had positive contact with COVID-19 patients during the study period, were all accounted for in the study. Categories of risk exposures of the affected HCWs were identified and assigned into high-risk exposure, medium-risk exposure, low-risk exposure, or no identifiable risk according to the Ministry of Health Malaysia Management of Healthcare Workers During COVID-19 Outbreak [9].

### Data analysis

The data was collected and tabulated according to four categories which were medical doctors, AMOs, nurses, and health care assistants on Microsoft Excel version 16.0 (Microsoft Corp. Redmond, WA). The data was analysed descriptively for exposure to the COVID-19 patients in terms of numbers, percentage, frequency, mean and standard deviations for the period before and after the restructuring.

## Results

There were 846 staff working in the ETDHKL during the study period of which 221 was medical doctors, 242 AMOs, 165 nurses and 228 health care assistants (Table 3). The department received a total of 20,495 visits whereby 14082 were prior to the changes and 6413 after. The inclusion criteria were all doctors, nurses, AMOs, and health care assistants working in the ETDHKL during the study period while the exclusion criteria were the clerks, administrative staff, and cleaners who do not come in contact with patients. Administrative staff were located in the administrative areas and away from clinical areas.

**Table 3:** Demography of staff category in ETDHKL

Staff category	***n*** (%)
Doctor	211 (24.9)
Assistant medical officers	242 (28.6)
Nurse	165 (19.5)
Health care assistant	228 (26.9)
Total number of Staff	846

Before the binary triage system and restructuring of the ETDHKL, there were 11 cases admitted from the ETDHKL with COVID-19 RT-PCR positive. Post restructuring, there were 10 positive cases reported. During the pre-restructuring period, the contact tracing of HCWs revealed that 542 out of 846 ETDHKL personnel were exposed to COVID-19 patients (Table 4) with 15 (1.8%) staff having no identifiable risk, 504 (59.6%) low risk, 15 (1.8%) medium risk and 8 (0.9%) high risk. In the post-restructuring period, a total of 122 (14.4%) HCWs were exposed. Eight (0.9%) were with no identifiable risk, 109 (12.9%) low risk, 0 (0%) medium risk, and only 5 (0.1%) high risk. There was a reduction in the number of exposed HCWs in all risk categories. Comparatively, post-restructuring, there was a 77.5% reduction in the number of exposed HCWs (542 vs 122).

**Table 4:** Number and percentage of healthcare workers exposed to COVID-19 patients

	***n*** (%)	
Category of risk exposure	Pre-restructuring	Post-restructuring
No identifiable risk	15 (1.8)	8 (0.9)
Low risk	504 (59.6)	109 (12.9)
Medium risk	15 (1.8)	0
High risk	8 (0.9)	5 (0.1)
Total number of exposed HCWs	542 (64.1)	122 (14.4)

The percentage of exposed HCWs in relation to the total number of ETDHKL patients were 3.85% pre-restructuring and 1.90% post-restructuring (Table 5). This translates to a 50.65% reduction of the risk of COVID-19 exposure to HCWs.

**Table 5:** Number and percentage of healthcare workers exposed to COVID-19 patients in relation with number of patients attending ETDHKL

	Pre-restructuring	Post-restructuring
Number of exposed HCWs	542	122
Number of Covid19 patients	11	10
Number of patients who attended ETDHKL	14082	6413
Percentage of exposed HCWs in relation with the number of patients (%)	3.85	1.90

## Discussion

Learning from the Italian experience in handling an overwhelming surge of suspected COVID-19 patients, the ETDHKL developed a binary triage system that categorized the treatment areas into dirty and clean areas [17, 18]. The new paradigm of the triage system included the assessment of potential infectivity together with the patient’s severity and subsequent zoning.

The reason for the restructuring of the triage system was due to the undifferentiated presentations of the COVID-19 infection. WHO suggested that up to 80% of the infected patients are asymptomatic [10]. Several studies showed that asymptomatic cases ranged from 50 to 75% [19, 20]. Gabriel Yan et al. [21] reported cases of patients with fever, thrombocytopenia and positive dengue serology tests which turned out to be false positive as investigations later revealed that these patients have significant chest radiograph findings and were tested positive for COVID-19 RT-PCR. Hospital in Bergamo Italy reorganized their ED and created “clean” and “dirty” pathways [17]. “Dirty” pathways were dedicated to patients with respiratory symptoms and temperatures of more than 37.5 °C while “clean” pathways were for other patients. However, the Italian system only differentiates those with objective evidence of fever with respiratory symptoms and thus may not be able to detect asymptomatic or patients with atypical presentations. Therefore, the system that ETDHKL developed is more robust in identifying suspected COVID-19 patients.

Early experience in Wuhan illustrated how a cluster outbreak occurred from a single index patient being mistakenly admitted to surgical services for abdominal pain. This resulted in a nosocomial spread to 10 healthcare workers and 4 other patients, all presenting with gastrointestinal symptoms of diarrhoea, nausea, and loss of appetite [22]. Hence, early detection is crucial to contain unnecessary high-risk exposure. A similar concept was applied in our ETDHKL, but with additional caution when assessing fever. We included patients reporting symptoms of fever/febrile illness 10 days prior and who met the high-risk epidemiological criteria. Our system also included the creation of CMSA to cater to stable symptomatic patients.

CMSA is a conjoint operation run by multidisciplinary teams headed by an emergency physician. It is configured by large tents and situated next to the ETDHKL. CMSA has the capability to receive a high number of patients and is designed to reduced cross-contamination. The features include a waiting area with physical distancing, dedicated areas for history taking, examination, and swab sampling. Coordination with the public health unit plays a crucial role for CMSA to function effectively. The establishment of a separate treatment facility is a strategy to preserve the hospital bed capacity and reduce congestion in the ED [23, 24].

The restructuring and reorganization of ETDHKL reduced the risks of COVID-19 exposure to HCW by 74.3%. The effective measure of dividing patients into high- and low-risk infective categories has helped HCWs to undertake proper precautions and increase their vigilance when treating any patients. A study by Lai et al. [4] in Tongji Hospital, Wuhan, has proven that HCWs who do not work at the frontlines (staffs working in general wards) have contracted a high number of COVID-19 infections due to the lack of protective measures and inadequate PPE. From this study, it is imperative to separate the two pools of patients to reduce the risk of COVID-19 infections. The reduced number of exposed HCWs in turn may reduce the number of HCWs needed for quarantine and thus sustaining adequate numbers of medical personnel in the ED. Officially, only 2 HCWs in the ETDHKL contracted COVID-19 from infected COVID-19 patients in April 2020. This represents 0.004% among ETDHKL’s HCW in April. Until the time of the write up of this manuscript, there were no further reported cases of HCWs in the ETDHKL contracting the COVID-19 disease from patients.

Bressan S et al. [25] reported only 18% of surveyed EDs endorsed periodic active surveillance of ED staff. In ETDHKL, the infection control committee actively conducts HCWs risk assessment in the department in line with the recommendation from WHO. The Infection Control committee was tasked to detect and identify HCWs at high risk of exposure to COVID-19, reinforce HCWs to self-monitor for fever and other symptoms, and to prescribe medical leave or put HCWs in home quarantine when ill. The binary triage system introduced in our centre helps in limiting the introduction and spread of COVID-19 within the ETDHKL by health care personnel. Our analysis showed a reduction of more than 50% of HCWs exposed to suspected COVID-19 patients. Importantly it also showed a reduction of exposed HCWs under medium and high-risk categories. Wallace DW et al. [26] advocated regular updates to the ED approach to COVID-19 infection following the latest information in the WHO guidelines. This was also in line with the reorganization of the structure of the ED to cater for the appropriate infection prevention and control.

Administrative staff such as the clerks who work at the registration counter or in the office were not included in this study. This is because they did not fit the criteria of HCWs and do not have direct contact with COVID-19-positive patients. There have been no departmental reports of administrative staff who were positive for COVID-19 infection or who have been exposed for more than 15 min to any suspected or confirm COVID-19 patients. Hospital cleaners were not included in the study as they are not ED staff and do not have any contact directly with any patients. They do have a theoretical risk of contracting COVID-19 infection however there is limited evidence that fomites contaminated with COVID-19 may lead to infection in this group [27].

### Limitations and recommendations

There are some limitations in our study. Firstly, this was an observational study with the involvement of only a single centre. It would be better if there were data obtained from other hospitals which also carried out similar triage system changes. A study comparing two centres that have applied different structural re-organization and different triage systems would be better in order to evaluate the impact of the restructuring, however ethically it can cause unnecessary exposure to HCWs. A longer duration of data collection that consists of more COVID-19 patients going through the ETDHKL would definitely show a clearer impact of the effects of restructuring. The ED itself is a multidisciplinary entity joined by radiology staff, hospital cleaners, clerks, and pharmacy staff. These groups are technical staff from other departments and do not fit the HCW criteria in this study. The inclusion criteria may be expanded in future studies and thus provide more robust data. Other measures of reducing infectivity from patients to HCWs and among HCW themselves apart from the new triaging system and restructuring of ED were not studied. The major aim of the study was to see the impact of the restructuring on the exposure of ED HCWs to COVID-19. Hospital personnel which were categorized as non-HCWs were not supposed to be in the dirty area if they follow the rules that were outlined. Therefore, they were excluded from the study based on that presumption.

The aim of the study was to see the impact of the binary triage system and departmental restructuring on the exposure of HCWs to COVID-19 and this study suggests that there may be some contribution to this reduction. It is important to note that there are other factors also contributing to the risk of exposure of the HCWs to the COVID-19 infection such as adherence to PPE, universal wearing of masks in hospitals, and swabbing procedures in the ED. The contributions of these variables require further studies in the future.

## Conclusion

From this study, there is a reduction of exposed HCW to COVID-19 patients in both the medium and high-risk groups. The binary triage system facilitated by the reorganization of the ETDHKL layout suggests a possible effective measure in reducing the exposure of COVID-19 infection to HCWs. With the new triaging concept, it does not only focus on the severity and signs of infectivity but also evolves further to fit the current needs of the health care system. The effectiveness of the new systems in the ETDHKL may hold the key in combating future outbreaks of infections and serves as the last line of defence against the spread of COVID-19 infections to HCWs.

## Data Availability

The data generated and analysed in this study are included in this article.

## Abbreviations

HCW: Health care worker
COVID-19: Coronavirus disease 2019
PPE: Personal protective equipment
SARI: Severe acute respiratory illness
WHO: World Health Organization
ED: Emergency department
ETDHKL: Emergency and Trauma Department, Hospital Kuala Lumpur
MOH: Ministry of Health
CMSA: COVID-19 mass screening area
ILI: Influenza-like illness
PUI: Patient under investigation
AMO: Assistant medical officer
SARS-CoV-2: Severe acute respiratory syndrome coronavirus 2
